# Understanding Girls' Motivation to Participate in Sport: The Effects of Social Identity and Physical Self-Concept

**DOI:** 10.3389/fspor.2021.787334

**Published:** 2022-01-11

**Authors:** Ross M. Murray, Alyona Koulanova, Catherine M. Sabiston

**Affiliations:** Faculty of Kinesiology and Physical Education, University of Toronto, Toronto, ON, Canada

**Keywords:** autonomous motivation, controlled motivation, self-perceptions, badminton, mediation

## Abstract

**Introduction:** Girls are often less motivated to participate in community sport compared to boys. Having a strong social identity with a sports team is positively associated with motivation to continue participation in sport, yet the mechanisms explaining this association are not well-known. In the current study, physical self-concept is tested as a mediator of the association between social identity and motivation.

**Method:** Girl badminton athletes were recruited to examine how the team environment shapes physical self-concept, and whether this association relates to motivation to participate in sport. Ninety-two girls completed a self-report survey to measure social identity, physical self-perceptions, and motivation. Two mediation models were conducted to examine whether physical self-concept mediated the relationship between social identity and autonomous motivation and controlled motivation.

**Results:** Physical self-concept partially mediated the relationship between social identity and autonomous motivation. The bootstrapped unstandardized indirect effect was, *b* = 0.05, 95% CI = 0.002 to.14. Physical self-concept fully mediated the relationship between social identity and controlled motivation. The bootstrapped unstandardized indirect effect was, *b* = −0.13, 95% CI = −0.30 to −0.01, *p* = 0.04.

**Discussion:** These results highlight the importance of the group context in relation to individual physical self-concept and motivation. Overall, targeting aspects of the team environment in community-level sport may be an important strategy to improve girls' physical self-concept, and autonomous motivation to continue sport participation.

Fewer girls participate in community sports compared to boys (Bélanger et al., 2009), and many girls drop out of sport during adolescence (Canadian Women and Sport, 2020). There are many reasons that explain why girls participate in sport at much lower rates than boys, including limited opportunities, a lack of representation and leadership opportunities, and societal pressure (Burton, 2015; Sabiston et al., 2019). For example, part of the gender participation gap in sport can be attributed to the female/athlete paradox, whereby the prototypical athletic body (e.g., muscular, powerful, strong) does not align with societal standards of the prototypical feminine body (e.g., tall, thin) (Krane et al., 2004). Lunde and Gattario (2017) noted that girls struggle to navigate this paradox within their self-concept, and negative perceptions of body image and physical self-concept have a demotivating influence on sport participation (Sabiston et al., 2019).

As a result of the struggles to straddle between athletic and feminine expectations (Krane et al., 2004; Sabiston et al., 2007), there have been many targeted campaigns worldwide aimed at dismantling harmful narratives around girls' participation in sport and physical education (Mulgrew et al., 2018). For example, campaigns such as “This Girl Can” in the United Kingdom, and “She's got it all” in Canada have brought attention to the gender participation gap. However, while many of these campaigns aim to shift societal perceptions around girls' participation in sport, they also place onus on girls and women to go out and get fit (Depper et al., 2019). Another approach to understanding girls sport and physical activity motivation is to examine the individual processes impacting motivation. For example, girls' participation in voluntary physical education classes often stems from their internal motivation (Lonsdale et al., 2011) and their close friends' participation (i.e., they want to participate with their friends) (Fitzgerald et al., 2012). This highlights the importance of the social environment to girls' decision making and indicates the importance of understanding more individual processes to develop more localized approaches that can impact girls' motivation in sport. Specifically, social factors are vital to girls' self-concept and motivation to participate in sport (McDonough and Crocker, 2005), and the team context can be a powerful factor that shifts individuals' perceptions of the self (Rees et al., 2015). In the current study, we examine whether feeling a strong sense of social identity with a sports team is related to girls' physical self-concept and motivation for sport.

It is important to consider the context in which girls participate in sport. Badminton is a global sport, and participation holds significant cultural meaning to many athletes (Chan and Lee, 2020). Although badminton is typically considered an individual sport, badminton athletes train and compete within team environments (Evans et al., 2012), whereby, athletes have training teammates, playing partners, coaches, and trainers who are considered part of their team. Community badminton teams in Canada are often composed of mixed genders, meaning girls and boys typically train together. As such, it is common for girls to have boys and men as coaches, partners, and opponents. These unique characteristics of the badminton environment make it an ideal sport to study social identity, self-concept, and motivation processes.

Drawing on multidimensional conceptualizations of the self, the self-concept encompasses many dimensions including the social self, emotional self, and the physical self, (Craven and Marsh, 2008). The physical self-concept is a particularly important facet of the self for adolescent girls and is associated with numerous health and well-being outcomes (Klomsten et al., 2004). In the sport context, girls' physical self-concept has been linked to greater sport commitment (deJonge et al., 2019), health behaviors such as dietary restraint and physical activity (Crocker et al., 2007), and motivation (Lohbeck et al., 2021). However, compared to boys, girls often report worse perceptions of their physical self-concept (Klomsten et al., 2004), which may have a demotivating effect on participation in physical activity and sport (Brunet et al., 2012). As such, improving girls' physical self-concept might be an effective strategy to help keep girls motivated to participate in sport through adolescence.

According to the social identity approach (Tajfel et al., 1971), individuals perceive themselves as part of social categories (e.g., a Canadian, a badminton player, a team member), which facilitates a sense of social belongingness and emotional attachment. This social identity can provide individuals with a sense of who they are, and can shape individuals' behavioral, affective, and cognitive processes. In sports, athletes can gain a strong sense of social identity from membership within their teams (Rees et al., 2015). Importantly, developing a social identity within a sports team can impact the self-concept. When individuals report high levels of social identity with an in-group (e.g., other athletes, team), they are more likely to report that traits relative to the in-group are also self-descriptive (Sim et al., 2014). This is because, according to the social identity approach, high levels of social identity minimize intra-group differences, and encourages group members to feel a sense of similarity with their in-group (Ellemers et al., 2004). In sport, this means athletes are more likely to describe themselves using traits believed to be descriptive of their team. For example, a girl who believes her sport team is strong and fast will be more likely to report herself being strong and fast. Therefore, in sports such as badminton, social identity can heighten perceptions of similarity within teams (Ellemers et al., 2004; Postmes et al., 2005) and gender differences related to physical self-concept within co-recreational teams are likely to be minimized. Further, beyond the known impacts of social identity on individual processes, badminton participants report that their participation has cultural significance to their identity and acts as a mechanism for self-expression (Chan and Lee, 2020). As such, examining the impact of social identity within badminton teams offers a unique opportunity to understand identity factors contributing to girls' physical self-concept and motivation.

Many conceptual models link social processes, identity, and motivation (White, 1959; Scanlan et al., 1993; Wigfield and Eccles, 2000). Understanding girls' motivational processes pertinent to sport participation is important to improving girls' adherence to sport, and their overall well-being. Based on self-determination theory (Deci and Ryan, 1985; Ryan and Deci, 2002) motivation is generally defined as autonomous (i.e., motivation stemming from engaging in sport because it is important, valued, enjoyable, and part of one's identity) or controlled (i.e., motivation stemming from pressure from others, avoidance of negative emotions such as shame and guilt). Autonomous motivation is typically associated with increased engagement in an exercise and physical activity (Brunet et al., 2012; Murray et al., 2021), whereas controlled forms of motivation are often associated with less engagement in physical activity and sport (Li et al., 2014).

According to Self-Determination Theory (Ryan and Deci, 2000), perceptions of competence, autonomy, and connectedness are believed to predict autonomous and controlled motivation. Further, social identity with one's team relates to more autonomous levels of motivation for sport (Vella et al., 2020) which, according to SDT, may relate to feelings of connectedness associated with social identity. Also, perceptions of physical self-concept likely relate to motivation as sport competence is an important component in athletes' physical self-concept (Marsh et al., 2010). These constructs take on heightened importance among girls in sport, as social factors play an important role in girls' self-concept and motivation (McDonough and Crocker, 2005).

Social identity and physical self-concept are important concepts likely underpinning the gender participation gap. Identifying whether these concepts relate to motivation could facilitate team level strategies aimed at improving motivation and reducing the gender participation gap. The purpose of the current study, therefore, was to examine associations between social identity, physical self-concept, and motivation in girl badminton athletes. Drawing on conceptual models of social processes, self and identity, and motivation (White, 1959; Deci and Ryan, 1985; Scanlan et al., 1993; Wigfield and Eccles, 2000), physical self-concept was proposed as a mediator of the relationship between social identity and motivation. It was hypothesized that high levels of social identity with a badminton team, and high levels of physical self-concept, would be associated with higher levels of autonomous motivation and lower levels of controlled motivation. Further, it was hypothesized that associations between social identity and motivation would be mediated by physical self-concept such that the indirect effect would be significant. This study was initiated through a partnership with Badminton Canada to explore participation factors among girl athletes.

## Method

### Participants and Procedure

Ethical approval was granted by an ethics review board at a Canadian University. Badminton Canada sent a study invitation to all contacts with emails within their database. Ninety-two girls completed all social identity, physical self-concept, motivation, and demographic items. Two follow-up reminder emails were sent two weeks apart. Participants 18 years of age or older provided informed consent, while participants under 18 years old provided assent and parental consent before completing the study. Upon providing assent or consent, participants completed an online questionnaire hosted on Survey Monkey. Questionnaires consisted of demographic items, measures assessing girls' social identity with their badminton team, perceptions for their physical self-concept, and motivation.

### Measures

#### Social Identity

Social identity was measured using the Social Identity Questionnaire for Sport (SIQS: (Bruner and Benson, 2018). The SIQS uses 9 items on a 7-point scale (1 strongly disagree, to 7 strongly agree) to assess social identity across three dimensions, in-group affect (e.g., In general, I'm glad to be a team member), cognitive centrality (e.g., In general, being a team member is an important part of my self-image), and in-group ties (e.g., I have a lot in common with other members in this team). Scores were averaged across all items for an overall social identity score ranging from 1 to 7, with higher scores indicating higher social identity. The SIQS has demonstrated good internal consistency and construct validity (Bruner et al., 2014; Bruner and Benson, 2018). In the current study, Cronbach's alpha was 0.93.

#### Global Physical Self-Concept

Physical self-concept was measured using two items from the Short Form Physical Self-Description Questionnaire (Marsh et al., 2010). Items were, “Physically, I am happy with myself”, and “Physically, I feel good about myself”. Items were rated on a 6-point scale from (1) false, to (6) true. These two items were correlated at *r* =0.93. Items were averaged together for an overall physical self-concept score ranging from 1 to 6, with higher scores indicating better physical self-concept.

#### Motivation

Motivation was measured using items from the Behavioral Regulation in Sport Questionnaire (Lonsdale et al., 2008). The stem for the measure was adjusted to represent motivation to participate in badminton specifically (e.g., I participate in badminton because…), and these items were rated on a 7-point scale from (1) not true at all to (7) very true. The items with high factor loadings and low standard errors for behavioral regulations specific to autonomous (identified regulation, and intrinsic motivation) and controlled (external regulation, introjected regulation) motivation were used (Viladrich et al., 2013). Recent research analyzing social identity and motivation in youth sport has used this abbreviated version of the Behavioral Regulation in Sport Questionnaire (Vella et al., 2020). The items were: “Because I feel pressure from other people to play” and “Because I would feel ashamed if I quit”. Items were correlated at *r* = 0.61 and a mean of the scores were generated for a total controlled motivation score ranging from 1 to 7 with higher scores representing higher levels of controlled motivation. Scores for the autonomous motivation items “Because I value the benefits of badminton” and “Because it's fun” were correlated at *r* = 0.51 and a mean of the scores was generated for a total autonomous motivation score ranging from 1 to 7, with higher scores indicating higher levels of autonomous motivation.

#### Demographics

Participants were asked to report demographic items including their age, the gender of their coach, and the number of years they have participated in badminton.

### Analyses

All analyses were conducted on R version 4.1.2 and mediation models were run using the Mediation package in R. Regression coefficients were used to examine direct relationships between study variables. To examine the hypothesis that the relationship between social identity and motivation is mediated through physical self-concept, we conducted a bootstrap mediation analyses with 1000 repetitions, consistent with recommendations by Hayes (2017). Statistical significance was indicated by *p* values less than 0.05 and 95% bias-corrected confidence intervals where the estimate of the indirect effect did not include zero.

## Results

Participants were adolescent girls and young adult women whose age ranged from 12 to 23 years (*N* = 92, *M*_age_ = 16.6, SD = 2.7). Participants ranged from having 1 to 11 years of experience playing badminton, with an average of 5.9 years in the sport. Sixty eight (74%) girls had a male as their primary coach and 24 reported a female primary coach. Means, standard deviations, and correlations between study variables are presented in Table 1. Overall, girls reported higher levels of autonomous motivation compared to controlled motivation. Girls' age did not significantly correlate with social identity, physical self-concept or autonomous and controlled motivation. Levels of social identity were significantly positively related to physical self-concept and autonomous motivation, but not controlled motivation. Autonomous motivation was negatively correlated with controlled motivation.

**Table 1:** Means, standard deviations, and correlations all study variables.

**Variable**	***M* (SD)**	**1**	**2**	**3**	**4**
1. Age	17.38 (2.99)				
2. Social Identity	4.95 (1.19)	0.12			
3. Physical Self-Concept	4.02 (1.48)	0.08	0.23^*^		
4. Autonomous Motivation	6.21 (0.94)	−0.06	0.40^**^	0.37^**^	
5. Controlled Motivation	3.38 (1.87)	0.03	−0.13	−0.37^**^	−0.42^**^

*M and SD are used to represent mean and standard deviation, respectively*.

* *indicates p < 0.05*.

** *indicates p < 0.01*.

### Autonomous Motivation

After controlling for participant age, regression coefficients indicated a significant positive relationship between social identity and autonomous motivation (*b* = 0.32, *SE* = 0.06, *p* < 0.01), and a significant positive relationship between social identity and physical self-concept (*b* = 0.28, *SE* = 0.10, *p* < 0.01). When accounting for the variance in age and social identity, physical self-concept was significantly correlated with autonomous motivation (*b* = 0.20, *SE* = 0.05, *p* < 0.01). The bootstrapped unstandardized indirect effect of social identity on autonomous motivation through physical self-concept was significant (*b* = 0.05, 95%CI = [0.002, 0.14], *p* = 0.04). The direct effect of social identity on autonomous motivation was also significant in the mediation model (*b* = 0.27, 95% CI = [0.13, 0.42], *p* < 0.01), indicating that the relationship between social identity and autonomous motivation was partially mediated through physical self-concept.

### Controlled Motivation

After controlling for participant age, regression coefficients indicated a non-significant negative association between social identity and controlled motivation (*b* = −0.21, *SE* = 0.16, *p* = 0.19). When accounting for the variance in age and social identity, physical self-concept was significantly negatively correlated with controlled motivation (*b* = −0.45, *SE* = 0.13, *p* < 0.01). The bootstrapped unstandardized indirect effect of social identity on controlled motivation through physical self-concept was significant (*b* = −0.13, 95%CI = [−0.30, −0.01], *p* = 0.04). The direct effect of social identity on controlled motivation was not significant in the mediation model (*b* = −0.08, 95% CI = [−0.38, 0.22], *p* = 0.54), indicating that the relationship between social identity and controlled motivation was mediated through physical self-concept.

## Discussion

Strategies are needed to increase girls' sport participation. In fact, Badminton Canada initiated a partnership to identify factors associated with girls' participation in the sport. Individuals who share high levels of social identity with their sport teams are typically more motivated to participate in sport (Vella et al., 2020), and motivation is critical to participation (Ullrich-French and Smith, 2009). Therefore, we investigated whether social identity relates to girls' motivation to participate in badminton, and whether physical self-concept can act as a mechanism within this relationship. Results of the current study provide evidence that badminton players who identify with their team report better perceptions of their physical self, which relates to higher levels of autonomous motivation.

Social identity and physical self-concept may relate to autonomous motivation because both concepts relate to internal constructs; while controlled motivation often derives from social processes external to the individual, such as a parent or coach (O'Neil and Hodge, 2020). For example, girls internalize their team membership to their own self-concept, shaping their perceptions of themselves as individuals (Rees et al., 2015); however, external pressures, such as an ego-oriented coach, would be more influential on controlled motivation. As such, strategies aimed at improving social identity and physical self-concept might help increase girls' autonomous motivation, but further research is needed to understand how external pressures can be targeted to reduce levels of controlled motivation.

The novel finding that social identity relates to girls' physical self-concept highlights the importance of the team dynamics within mixed-gendered sport. Physical self-perceptions are an important part of athletes' sport experiences (Sabiston et al., 2019), and this study indicates that the teams athletes participate on relates to their physical self-perceptions. Specifically, encouraging athletes to feel a sense of social identity with their team might help enhance their physical self-concept. Typically, individuals think more positively about their groups than they do themselves (Cruwys et al., 2015), and the current study indicates that at higher levels of social identity, positive perceptions of the group might translate to the self. The processes that directly link social identity and physical self-concept, and the longitudinal nature of the associations, require further investigation.

The current research also highlights the importance of understanding associations between community sport team processes and individual processes. Specifically, researchers and policy makers should consider team level strategies aimed at keeping girls in sport. Adopting a social identity approach (Haslam et al., 2016) to improve perceptions of self-concept can be implemented at the team level through coaches and sport administrators. For example, focusing on social identity leadership, which includes running workshops through team leaders to develop a sense of “us” among team athletes (Haslam et al., 2017; Slater and Barker, 2019), may encourage recreational girl athletes to identify with their teams. This in turn may be an effective strategy to reach a wide range of athletes, to shift perceptions of physical self-concept throughout sport.

Social identity processes can also expand beyond the sport team level, to the extent athletes identify with their community, sport, nation, and culture (Rees et al., 2015). Current results indicate social identity related to one's badminton team correlates with physical self-concept and motivational processes; however, further research is needed to understand whether social identity at other levels (e.g., culture) can influence perceptions of the self and motivation to participate in sport. For example, badminton athletes in Hong Kong report badminton can act as a mechanism to connect with cultural roots (Chan and Lee, 2020). This indicates participation in badminton relates to cultural and national identity processes; how these processes relate to self-perceptions and sustained participation in the sport may reveal strategies for improving the community sport environment, especially in countries where badminton is not considered a primary sport.

There are some limitations in the current study that should be addressed in future research. Due to the cross-sectional design, causal inferences cannot be made. Future research is needed to examine the observed associations using a larger sample across a season, and longitudinal associations. Also, because email study advertisements were sent to those within the Badminton Canada database (not only eligible participants), the total response rate is unknown. Further, abbreviated measures of physical self-concept and motivation regulations were used. Although these items are believed to be representative of the constructs, further research is needed to test these associations using full measures of global physical self-concept and controlled and autonomous motivation. While autonomous and controlled motivation were explored in this community sport sample, future research efforts may be needed to expand upon the associations with social identity, physical self-concept, and the unique behavioral regulations used to operationalize motivation (e.g., external, introjected, identified, integrated, internal). Finally, the competition level or ranking of athletes were not accounted for. These factors might influence girls' perceptions of physical self-concept and the extent to which they identify with their team.

It is important to note that girls and women in our sample trained and competed within mixed gender teams. While the results of this study should be tested within single-gender teams, the extent to which mixed gender teams impact girls identity, self-concept, and motivation poses an interesting research question. Outside sport, the effect of single-gender physical education classes have had mixed results for girls' experiences (McKenzie et al., 2004; Fairclough and Stratton, 2006). From a social identity approach (Rees et al., 2015), levels of social identity might moderate the association between gender differences and girls' experiences within community sport, whereby, high levels of social identity minimize perceived gender differences within a team, leading to better mixed-gender sport experiences. However, this hypothesis needs to be explored through future research.

Limitations notwithstanding, the current findings and previous research highlight the importance of self-perceptions within girls' sport motivation and participation (Sabiston et al., 2019). It is important that leaders in sport develop strategies at the organizational level to develop a sport environment that supports positive perceptions of the physical self. Specifically, facilitating social identity within sport teams might be an effective strategy to improve girls' physical self-concept and autonomous motivation to participate in sport. Social identity leadership can be used as a mechanism to increase social identity within sport teams (Slater and Barker, 2019). This leadership strategy involves coaches and/or leaders identifying important identities within their teams and clarifying goals and aspirations in line with team identities (Haslam et al., 2017). Overall, further research is needed to develop and test specific strategies that can improve the team environment to help keep girls motivated to participate in sport.

## Data Availability Statement

The raw data supporting the conclusions of this article will be made available by the authors, without undue reservation.

## Ethics Statement

The studies involving human participants were reviewed and approved by Social Sciences, Humanities and Education REB. Written informed consent to participate in this study was provided by the participants' legal guardian/next of kin.

## Author Contributions

RM was responsible for the conceptualization, data curation, analysis and writing, reviewing, and editing of the manuscript. AK was responsible for conceptualization, data curation, writing, reviewing, and editing. CS was responsible for funding acquisition, conceptualization, and writing and editing.

## Funding

This work was funded with a partnership support of a SIRC Match Grant.

## Conflict of Interest

The authors declare that the research was conducted in the absence of any commercial or financial relationships that could be construed as a potential conflict of interest.

## Publisher's Note

All claims expressed in this article are solely those of the authors and do not necessarily represent those of their affiliated organizations, or those of the publisher, the editors and the reviewers. Any product that may be evaluated in this article, or claim that may be made by its manufacturer, is not guaranteed or endorsed by the publisher.

## References

[B1] Bélanger M. Gray-Donald K. O'Loughlin J. L. Paradis G. Hanley J. (2009). When adolescents drop the ball sustainability of physical activity in youth. Am. J. Prevent. Med. 37, 41–49. 10.1016/j.amepre.2009.04.002 19524143

[B2] Bruner M. W. Benson A. J. (2018). Evaluating the psychometric properties of the Social Identity Questionnaire for Sport (SIQS). Psychol. Sport Exer. 35, 181–188. 10.1016/j.psychsport.2017.12.006

[B3] Bruner M. W. Boardley I. D. Côté J. (2014). Social identity and prosocial and antisocial behavior in youth sport. Psychol. Sport Exer. 15, 56–64. 10.1016/j.psychsport.2013.09.003 29058105

[B4] Brunet J. Sabiston C. M. Castonguay A. L. Ferguson L. Bessette N. (2012). The association between physical self-discrepancies and women's physical activity: The mediating role of motivation. J. Sport Exer. Psychol. 34, 102–123. 10.1123/jsep.34.1.102 22356878

[B5] Burton L. J.. (2015). Underrepresentation of women in sport leadership: a review of research. Sport Manage. Rev. 18, 155–165. 10.1016/j.smr.2014.02.004

[B6] Canadian Women and Sport. (2020). The Rally Report: Encouraging Action to Improve Sport for Women and Girls. June 7.

[B7] Chan B. C. L. Lee B. (2020). Wellbeing and personality through sports: a qualitative study of older badminton players in two cultures. Qualit. Res. Sport Exer. Health. 12, 350–362. 10.1080/2159676X.2019.1606850

[B8] Craven R. G. Marsh H. W. (2008). The centrality of the self-concept construct for psychological wellbeing and unlocking human potential: Implications for child and educational psychologists. Educ. Child Psychol. 25, 104–118. Available online at: https://psycnet.apa.org/record/2009-06944-010

[B9] Crocker P. R. E. Sabiston C. M. Kowalski K. C. McDonough M. Kowalski N. (2007). Longitudinal assessment of the relationship between physical self-concept and health-related behavior and emotion in adolescent girls. J. Appl. Sport Psychol. 18, 185–200. 10.1080/10413200600830257

[B10] Cruwys T. South E. I. Greenaway K. H. Haslam S. A. (2015). Social identity reduces depression by fostering positive attributions. Soc. Psychol. Personal. Sci. 6, 65–74. 10.1177/1948550614543309

[B11] Deci E. L. Ryan R. M. (1985). Intrinsic Motivation and Self-Determination in Human Behavior. Plenum Publishing Co. 10.1007/978-1-4899-2271-7

[B12] deJonge M. Mackowiak R. Pila E.va. Crocker P. R. E. Sabiston C. M. (2019). The relationship between sport commitment and physical self-concept: Evidence for the self-enhancement hypothesis among adolescent females. J. Sports Sci. 37 2459–2466. 10.1080/02640414.2019.1641381 31288678

[B13] Depper A. Fullagar S. Francombe-Webb J. (2019). This girl can? The limitations of digital do-it-yourself empowerment in women's active embodiment campaigns. In: Digital Dilemmas. 10.1007/978-3-319-95300-7_9

[B14] Ellemers N. de Gilder D. Haslam S. A. (2004). Motivating individuals and groups at work: A social identity perspective on leadership and group performance. Acad. Manage. Rev. 29, 459–478. 10.2307/20159054

[B15] Evans M. B. Eys M. A. Bruner M. W. (2012). Seeing the “we” in “me” sports: The need to consider individual sport team environments. Canad. Psychol. 53, 301–308. 10.1037/a0030202

[B16] Fairclough S. J. Stratton G. (2006). Effects of a physical education intervention to improve student activity levels. Phys. Educ. Sport Pedag. 11, 29–44. 10.1080/17408980500467613

[B17] Fitzgerald A. Fitzgerald N. Aherne C. (2012). Do peers matter? A review of peer and/or friends' influence on physical activity among American adolescents. J. Adolescence. 35, 941–958. 10.1016/j.adolescence.2012.01.002 22285398

[B18] Haslam C. Cruwys T. Haslam S. A. Dingle G. Chang M. X. (2016). GROUPS 4 HEALTH: Evidence that a social-identity intervention that builds and strengthens social group membership improves mental health. J. Affect. Disord. 194, 188–195. 10.1016/j.jad.2016.01.010 26828756

[B19] Haslam S. A. Steffens N. K. Peters K. Boyce R. A. Mallett C. J. Fransen K. (2017). A social identity approach to leadership development; The 5R program. J. Person. Psychol. 16, 113–124. 10.1027/1866-5888/a000176

[B20] Hayes A. F.. (2017). Introduction to Mediation, Moderation, and Conditional Process Analysis: A Regression-Based Approach. Guilford publications.

[B21] Klomsten A. T. Skaalvik E. M. Espnes G. A. (2004). Physical self-concept and sports: do gender differences still exist? Sex Roles. 50, 119–127. 10.1023/B:SERS.0000011077.10040.9a

[B22] Krane V. Choi P. Y. L. Baird S. M. Aimar C. M. Kauer K. J. (2004). Living the paradox: female athletes negotiate femininity and muscularity. Sex Role. 50, 315–329. 10.1023/B:SERS.0000018888.48437.4f

[B23] Li K. Iannotti R. J. Haynie D. L. Perlus J. G. Simons-Morton B. G. (2014). Motivation and planning as mediators of the relation between social support and physical activity among U.S. adolescents: a nationally representative study. Int. J. Behav. Nutrit. Phys. Activity. 11, 1–9. 10.1186/1479-5868-11-42 24656181 PMC3994425

[B24] Lohbeck A. von Keitz P. Hohmann A. Daseking M. (2021). Children's physical self-concept, motivation, and physical performance: does physical self-concept or motivation play a mediating role? Front. Psychol. 12, 1185. 10.3389/fpsyg.2021.669936 33995228 PMC8121452

[B25] Lonsdale C. Hodge K. Rose E. A. (2008). The Behavioral Regulation in Sport Questionnaire (BRSQ): Instrument development and initial validity evidence. J. Sport. Exer. Psychol. 30, 323–355. 10.1123/jsep.30.3.323 18648109

[B26] Lonsdale C. Sabiston C. M. Taylor I. M. Ntoumanis N. (2011). Measuring student motivation for physical education: Examining the psychometric properties of the Perceived Locus of Causality Questionnaire and the Situational Motivation Scale. Psychol. Sport Exer. 12, 284–292. 10.1016/j.psychsport.2010.11.003

[B27] Lunde C. Gattario K. H. (2017). Performance or appearance? Young female sport participants' body negotiations. Body Image. 21, 81–89. 10.1016/j.bodyim.2017.03.001 28365534

[B28] Marsh H. W. Martin A. J. Jackson S. (2010). Introducing a short version of the physical self description questionnaire: new strategies, short-form evaluative criteria, and applications of factor analyses. J. Sport. Exer. Psychol. 32, 438–482. 10.1123/jsep.32.4.438 20733208

[B29] McDonough M. H. Crocker P. R. E. (2005). Sport participation motivation in young adolescent girls: The role of friendship quality and self-concept. Res. Quart. Exer. Sport. 76, 456–467. 10.1080/02701367.2005.10599319 16739684

[B30] McKenzie T. L. Prochaska J. J. Sallis J. F. LaMaster K. J. (2004). Coeducational and single-sex physical education in middle schools: Impact on physical activity. Res. Quart. Exer. Sport. 75, 446–449. 10.1080/02701367.2004.10609179 15673045

[B31] Mulgrew K. E. McCulloch K. Farren E. Prichard I. Lim M. S. C. (2018). This girl can #jointhemovement: Effectiveness of physical functionality-focused campaigns for women's body satisfaction and exercise intent. Body Image. 24, 26–35. 10.1016/j.bodyim.2017.11.007 29253826

[B32] Murray R. M. Howe H. S. Sylvester B. D. Willson E. Sabiston C. M. (2021). Associations between resistance training motivation, behaviour and strength. Int. J. Sport. Exer. Psychol. 10.1080/1612197X.2021.1929400. [Epub ahead of print].

[B33] O'Neil L. Hodge K. (2020). Commitment in sport: the role of coaching style and autonomous versus controlled motivation. J. Appl. Sport Psychol. 32, 607–617. 10.1080/10413200.2019.1581302

[B34] Postmes T. Haslam S. A. Swaab R. I. (2005). Social influence in small groups: an interactive model of social identity formation. Eur. Rev. Soc. Psychol. 16, 1–42. 10.1080/10463280440000062

[B35] Rees T. Haslam S. A. Coffee P. Lavallee D. (2015). A social identity approach to sport psychology: principles, practice, and prospects. Sport Med. 45, 1083–1096. 10.1007/s40279-015-0345-4 26092187

[B36] Ryan R. M. Deci E. L. (2000). Self-determination theory and the facilitation of intrinsic motivation, social development, and well-being. Am. Psychol. 55, 68–78. 10.1037/0003-066X.55.1.68 11392867

[B37] Ryan R. M. Deci E. L. (2002). An overview of self-determination theory. In: Deci, E. L., and Ryan, R. M. (Eds.), Handbook of self-determination Res. pp. 3–33. University of Rochester Press.

[B38] Sabiston C. M. Pila E.va. Vani M. F. Thogersen-Ntoumani C. (2019). Body image, physical activity, and sport: A scoping review. Psychol. of Sport. Exer. 42, 48–57. 10.1016/j.psychsport.2018.12.010

[B39] Sabiston C. M. Sedgwick W. A. Crocker P. R. E. Kowalski K. C. Mack D. E. (2007). Social physique anxiety in adolescence: an exploration of influences, coping strategies, and health behaviours. J. Adolesc. Res. 22, 78–101. 10.1177/0743558406294628

[B40] Scanlan T. K. Carpenter P. J. Simons J. P. Schmidt G. W. Keeler B. (1993). The sport commitment model: Measurement development for the youth-sport domain. J. Sport. Exer. Psychol. 15, 16–38. 10.1123/jsep.15.1.16

[B41] Sim J. J. Goyle A. McKedy W. Eidelman S. Correll J. (2014). How social identity shapes the working self-concept. J. Exper. Soc. Psychol. 55, 271–277. 10.1016/j.jesp.2014.07.015

[B42] Slater M. J. Barker J. B. (2019). Doing social identity leadership: Exploring the efficacy of an identity leadership intervention on perceived leadership and mobilization in elite disability soccer. J. Appl. Sport Psychol. 31, 65–86. 10.1080/10413200.2017.1410255

[B43] Tajfel H. Billig M. G. Bundy R. P. Flament C. (1971). Social categorization and intergroup behaviour. European J. Soc. Psychol. 1, 149–178. 10.1002/ejsp.242001020225855820

[B44] Ullrich-French S. Smith A. L. (2009). Social and motivational predictors of continued youth sport participation. Psychol. Sport. Exer. 10, 87–95. 10.1016/j.psychsport.2008.06.007

[B45] Vella S. A. Benson A. J. Sutcliffe J. Mclaren C. Schweickle M. J. Miller A.. (2020). Self-determined motivation, social identification and the mental health of adolescent male team sport participants. J. Appl. Sport Psychol. 33, 452–466. 10.1080/10413200.2019.1705432

[B46] Viladrich C. Appleton P. R. Quested E. Duda J. L. Alcaraz S. Heuzé J. P.. (2013). Measurement invariance of the Behavioural Regulation in Sport Questionnaire when completed by young athletes across five European countries. Int. J. Sport. Exer. Psychol. 11, 384–394. 10.1080/1612197X.2013.830434

[B47] White R. W.. (1959). Motivation reconsidered: the concept of competence. Psychol. Rev. 66, 297–333. 10.1037/h0040934 13844397

[B48] Wigfield A. Eccles J. S. (2000). Expectancy – value theory of achievement motivation. Contemp. Educ. Psychol. 81, 68–81. 10.1006/ceps.1999.1015 10620382

