# Supplementary material for: Understanding Girls' Motivation to Participate in Sport: The Effects of Social Identity and Physical Self-Concept
Source: Front Sports Act Living. 2022 Jan 11;3:787334. doi: 10.3389/fspor.2021.787334 (PMC8787279; doi:10.3389/fspor.2021.787334)
Supplement: Supplementary file 1 [file Table_1.DOCX]

**Badminton Questionnaire items**

**Demographics**

Do you currently participate in badminton? Yes No

How old are you?

How do you identify? Male Female Other

How many years did you participate in badminton? ___ years

Is your main badminton coach? Man Woman Other

**Motivation**

| Select the number that most applies to how you feel.  1 = not at all true, 4 = Somewhat true, 7 = Very true | |
| --- | --- |
| Because it’s fun |  |
| Because I value the benefits of badminton |  |
| Because I would feel ashamed if I quit |  |
| Because I feel pressure from other people to play |  |

**Physical self-concept**

| Please indicate your agreement with the following statements:  1 = false; 2 = Mostly false; 3 = More false than true; 4 More true than false; 5 = Mostly true; 6 = true | |
| --- | --- |
| I often do exercise or activities that make me breathe hard |  |
| I do physically active things (e.g. jog, dance, bicycle, aerobics, gym, swim) at least three times per week |  |
| I do lots of sports, dance, gym, or other physical activities |  |
| I do sports, exercise, dance or other physical activities almost every day |  |
| I have a nice looking face |  |
| I am better looking than most of my friends |  |
| I am good looking |  |
| My waist is too large |  |
| I have too much fat on my body |  |
| I am overweight |  |
| I can run a long way without stopping |  |
| I can be physically active for a long period of time without getting tired |  |
| I am good at endurance activities e.g. distance running, aerobics, swimming, cross-country, skiing |  |
| Physically, I am happy with myself |  |
| Physically, I feel good about myself |  |
| I’m good at all sport |  |
| I have good sport skills |  |
| I play sports well |  |

**Social identity**

| Please click a number from 1 to 7 to indicate your agreement with each of the statements.  *1 = Strongly Disagree; 2 = Disagree; 3 = Slightly Disagree; 4 = Neither Agree nor Disagree; 5 = Slightly Agree; 6 = Agree; 7 = Strongly Agree* | |
| --- | --- |
| I feel strong ties to other members in this team |  |
| I find it easy to form a bond with other members in this team |  |
| I feel a sense of being “connected” with other members in this team |  |
| Overall, being a team member has a lot to do with how I feel about myself |  |
| In general, being a team member is an important part of my self-image |  |
| The fact that I am a team member often enters my mind |  |
| In general, I’m glad to be a team member |  |
| I feel good about being a team member |  |
| Generally, I feel good when I think about myself as a team member |  |
