# Supplementary material for: Understanding Girls' Motivation to Participate in Sport: The Effects of Social Identity and Physical Self-Concept
Source: Front Sports Act Living. 2022 Jan 11;3:787334. doi: 10.3389/fspor.2021.787334 (PMC8787279; doi:10.3389/fspor.2021.787334)
Supplement: Supplementary file 2 [file Data_Sheet_1.PDF]

Badminton Questionnaire items

Demographics

Do you currently participate in badminton? Yes No
How old are you?
How do you identify? Male Female Other
How many years did you participate in badminton? ___ years
Is your main badminton coach? Man Woman Other

Motivation

Select the number that most applies to how you feel.<br>1 = not at all true, 4 = Somewhat true, 7 = Very true
Because it's fun
Because I value the benefits of badminton
Because I would feel ashamed if I quit
Because I feel pressure from other people to play

Physical self-concept

Please indicate your agreement with the following statements:<br>1 = false; 2 = Mostly false; 3 = More false than true; 4 More true than false; 5 = Mostly true; 6 = true
I often do exercise or activities that make me breathe hard
I do physically active things (e.g. jog, dance, bicycle, aerobics, gym, swim) at least three times per week
I do lots of sports, dance, gym, or other physical activities
I do sports, exercise, dance or other physical activities almost every day
I have a nice looking face
I am better looking than most of my friends
I am good looking
My waist is too large
I have too much fat on my body
I am overweight
I can run a long way without stopping
I can be physically active for a long period of time without getting tired
I am good at endurance activities e.g. distance running, aerobics, swimming, cross-country, skiing
Physically, I am happy with myself
Physically, I feel good about myself
I'm good at all sport
I have good sport skills
I play sports well

Social identity

Please click a number from 1 to 7 to indicate your agreement with each of the statements.

1 = Strongly Disagree; 2 = Disagree; 3 = Slightly Disagree; 4 = Neither Agree nor Disagree; 5 = Slightly Agree; 6 = Agree; 7 = Strongly Agree
I feel strong ties to other members in this team
I find it easy to form a bond with other members in this team
I feel a sense of being “connected” with other members in this team
Overall, being a team member has a lot to do with how I feel about myself
In general, being a team member is an important part of my self-image
The fact that I am a team member often enters my mind
In general, I’m glad to be a team member
I feel good about being a team member
Generally, I feel good when I think about myself as a team member
